# “Pocket-sized RNA-Seq”: A Method to Capture New Mature microRNA Produced from a Genomic Region of Interest

**DOI:** 10.3390/ncrna1020127

**Published:** 2015-07-03

**Authors:** Florent Hubé, Claire Francastel

**Affiliations:** 1 University Paris Diderot, Sorbonne Paris Cité, 75205 Paris, France; 2 Epigénétique et Destin Cellulaire, CNRS UMR 7216, 75013 Paris, France

**Keywords:** RNA pull down, RACE-PCR, hsa-miR-21, hsa-miR-1237, mirtron, miRNA, mature miRNA sequence

## Abstract

Currently, the discovery of new small ncRNAs requires high throughput methods even in the case of focused research on the regulation of specific genes or set of genes. We propose herein a simple, rapid, efficient, and cost effective method to clone and sequence single, yet unknown, small ncRNA. This technique that we called “Pocket-sized RNA-Seq” or psRNA-seq is based on *in vitro* transcription, RNA pull down and adapted RACE-PCR methods that allow its implementation using either available commercial kits or in-house reagents.

## 1. Introduction

Small non-coding RNAs (ncRNAs), so named because of their size arbitrarily set below 200 nt, form a heterogeneous family of molecules holding important regulatory functions that have fundamentally transformed our understanding of gene regulatory networks [1,2]. Prominent examples include small nuclear RNA (snRNA) implicated in splicing events, small nucleolar RNA (snoRNA), small Cajal body RNA (scaRNA) and guide RNA (gRNA) implicated in RNA editing and modifications and microRNAs (miRNAs) that collectively regulate the expression of a large number of messenger RNAs (mRNAs) in a posttranscriptional manner, by either promoting their destabilization, repressing their translation, or both. They have attracted much attention owing to their major role in shaping the transcriptome and proteome, and ultimately the fate of eukaryotic cells.

In recent years, great efforts have been devoted towards the systematic identification of novel miRNAs and their target mRNAs, as well as their origin and biogenesis.

The biogenesis of miRNAs is a multi-step process; miRNA genes are transcribed by RNA polymerase II, giving rise to long primary miRNAs (pri-miRNAs) that are cleaved in the nucleus by the enzymes Drosha/DGRC8 of the microprocessor complex to produce a 70/100 nucleotide-long precursor (pre-miRNA) and then transported into the cytoplasm by Exportin 5 (for review see [3,4,5]). In the cytoplasm, pre-miRNAs are further processed by a Dicer into 17–25 nt-long mature miRNAs and loaded into the RNA-induced silencing complex (RISC). Typically, one of the two strands is preferentially selected by the RISC complex to bind the 3′-untranslated (3′-UTR) region of target genes, while the other one is degraded. In most cases, the consequence of a miRNA binding to its target gene is the mRNA degradation and/or inhibition of protein translation [3,4,5], although there have been reports of miRNAs stabilizing their target transcripts [6,7].

Most miRNAs are encoded by independent transcription units [8], located in intergenic regions or hosted within introns of protein-coding or non-coding genes [9,10,11,12,13]. Beside these intronic miRNAs that are potentially transcribed independently of the host genes, novel classes of intronic miRNAs precursors have been reported recently by which production is initiated by splicing of the intron, independently of the microprocessor [10,11,14]. This class currently consists of mirtrons—thereafter subdivided into canonical, 3′ and 5′ tailed that require exosome-mediated trimming after splicing—and simtrons—that require splicing and Drosha but not DGCR8—inherently co-regulated with their host gene and by alternative splicing of the intron [10,15].

To date, the only ways of studying mature miRNAs are (1) with high-throughput methods, which allows the sequencing of any RNAs detected in a cell; or (2) small-scale approaches, such as stem-loop RT-qPCR that all require to know the exact sequence of the mature miRNA.

Current methods of large-scale profiling of miRNAs include miRNA microarrays, bead-based capture methods, NanoString Technologies^®^, Taqman-based arrays or high-throughput sequencing. However, because deep sequencing-based expression analysis produces huge amounts of sequence data, there are substantial bioinformatic challenges to appropriately analyze and handle such sequence information. Another important consideration is the difficultly to identify miRNAs that are expressed at low levels, at highly specific stages of development, or in rare cell types. It is indeed well established that low read counts yielded by low expressing sequences show poor consistency between technical replicates, and exclusion of sequences with read counts less than a low threshold in each sample is a common strategy used to eliminate sequencing errors [16]. In principle, these limitations can be overcome by increasing the sequencing depth on small RNA libraries using a broad range of samples, although the challenge of huge data sets to be analyzed still remains. Additional limitations include sequence-specific biases related to the enzymatic steps used in the preparation of small RNA cDNA libraries that favor the capture of some miRNAs to the detriment of others [17,18,19].

In essence, despite rapid advancement in sequencing technologies and because of the general excitement surrounding miRNA discoveries and their potential therapeutic applications in numerous diseases, additional tools are required to enable researchers to accurately identify and study novel miRNAs when sensitivity on low-abundant miRNAs is an issue or bioinformatical analysis of substantial data represents a challenge. For these reasons, we propose herein a method to systematically identify, clone, and sequence unique unknown small ncRNAs, using miRNA as a proof of principle, from a genomic region of interest, that we named Pocket-sized small RNA-Seq (psRNA-seq) as a wink to the tiny sequencing reads it requires.

## 2. Results

**Figure 1:**
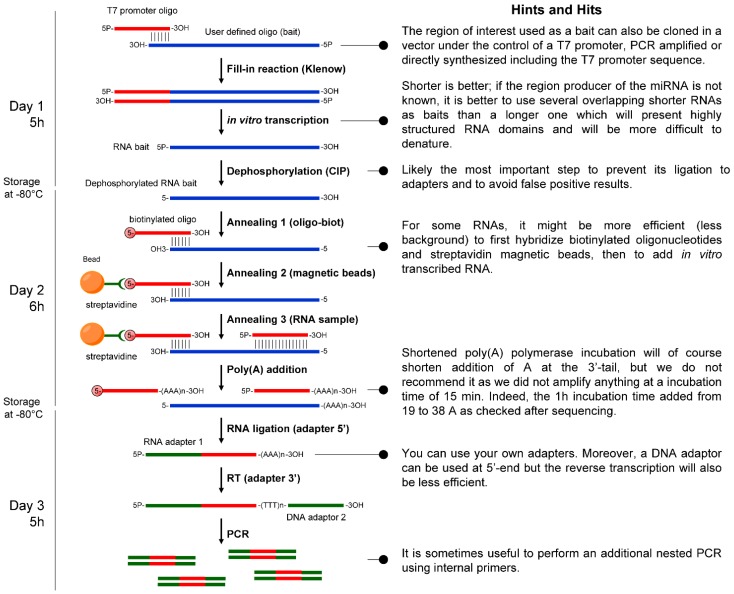
Scheme of the pocket-sized RNA-seq method. 5P-, 5′-phosphate group; 3OH-, 3′-hydroxyl group; 5-, dephosphorylated 5′-end; (AAA)n-3OH, polyadenylated 3′-end; (AAA)n and (TTT)n, polyA and polyT sequences, respectively; RACE, Rapid amplification of cDNA ends; RT, reverse transcription; CIP, calf intestinal phosphatase; oligo-biot, oligonucleotide biotinylated in 5′-end.

Detailed experimental protocol is given in Supplementary Materials and a scheme of the pocket-sized RNA-seq method is shown in Figure 1.

**Figure 2:**
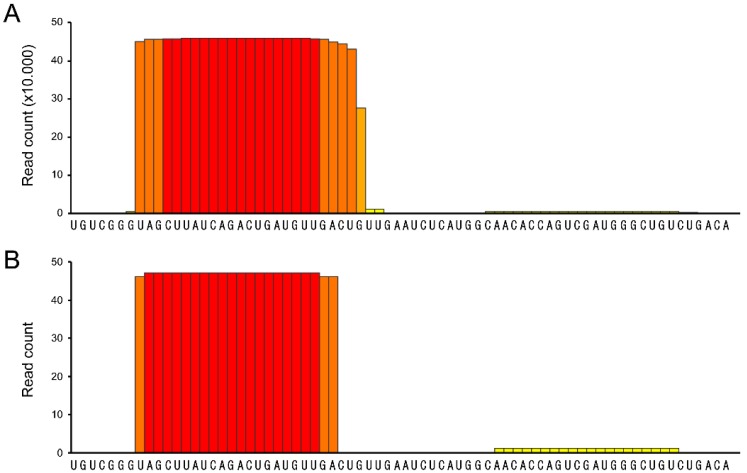
(**A**) Schematic representation of the 528.922 reads count corresponding to hsa-miR-21 according to miRBase release 21; (**B**) Schematic representation of the result of the 47 plasmids sequenced. Out of the 47 clones, 46 were corresponding to the guide mature miR-21 and one was related to the star mature miR-21 strand. Red bars represent 100% of presence of the nucleotide bellow, while yellow bars represent less than 5% of presence. Orange is intermediate percentage.

As a proof of principle for the efficiency of psRNAS-seq method in identifying miRNAs produced by a genomic region of interest, we used three 60–90 nt long sequences surrounding the precursor of hsa-miR-21 and proceeded as described in the Material and Methods section. Out of the 75 clones that were sequenced (25 per each three baits), 47 showed a hundred percent match with the mature hsa-miR-21 sequence (Figure 2) as referenced by miRBase website. More specifically, 25/25 clones contained the full sequence of the mature guide sequence of hsa-miR-21 pulled down using bait #1 and 21/25 using bait #2. One clone out of 25 contained the full sequence of the mature star strand of hsa-miR-21 that was pulled down using bait #2. All the other clones were empty (3/25 and 25/25 from bait #2 and bait #3 experiments, respectively). These data perfectly match miRBase data along which the ratio between reads mapping the guide and the star sequences is about 99.5% [20,21,22] while we obtained a ratio of 98% using our method. It is worth mentioning that even though the mature hsa-miR-21 star strand is highly under-represented according to miRBase, our developed psRNA-seq was sensitive enough to detect and clone it from a small number of colonies.

The presence of hsa-miR-21 in the sample material was also verified by stem-loop RT-qPCR [23], which can be developed only if the mature miRNA sequence is known. As shown in Figure S1A, mature hsa-miR-21 and hsa-miR-10a are indeed amplified from RNA samples, but only when RNAs were size-fractionated prior to stem-loop RT-qPCR amplification. Indeed, using stem-loop RT-qPCR (Figure S1B), the mature guide miR-21 strand was detected earlier from the small RNAs fraction (Ct 19.21 ± 1.01) than from the long RNAs fraction or total RNAs (Ct 27.15 ± 1.27 and 28.92 ± 0.74, respectively). No detection below the decisive 35 cycles was observed when using (i) random primers instead of stem-loop primers, (ii) specific primers for the star strand, (iii) primary myoblasts or control conditions omitting the RT (Figure S1B). While further studies are needed, we can surmise from these data that psRNA-seq method is likely to be more sensitive than the classical stem-loop RT-qPCR since only the former allowed detection of the minority hsa-miR-21 star strand in cancer cells.

We originally selected the well-characterized hsa-miR-21 as a proof of principle for the efficiency of psRNA-seq since hsa-miR-21 is a well-known oncomiR *i.e.*, a miRNA highly expressed in cancer cells and not at all or at very low levels in normal cells. We carried out the psRNA-seq method using sample RNAs isolated from cells of the breast cancer line MCF-7 or from human primary myoblasts and indeed verified that mature hsa-miR-21 was only captured from cancer MCF-7 cells (Figure S1C). This is in agreement with Burge’s small RNA-seq data using various cell lines and primary tissues (Figure S2A and S2B) showing that levels of hsa-miR-21 were much higher in transformed cell lines, especially in MCF-7 cells, than in muscle tissue (Figure S2A).

**Figure 3:**
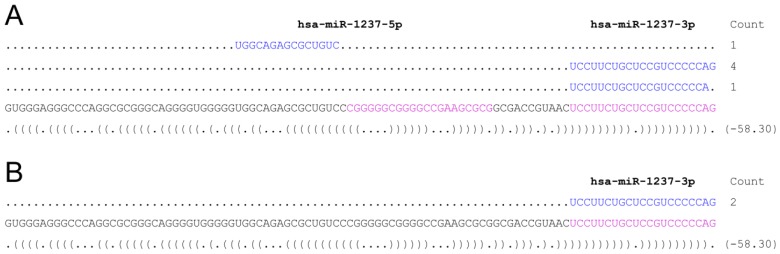
(**A**) Deep sequencing reads for stem-loop sequence MI0006327 corresponding to hsa-miR-1237 precursor according to miRBase release 21; (**B**) Sequencing read counts for stem-loop sequence hsa-miR-1237 precursor following our protocol. Two clones were sequenced which corresponded to the 3p-form of hsa-miR-1237.

In addition, we decided to use our method to identify a miRNA of mirtron origin, hsa-miR-1237 [24], processed from intron 11 (GRCh37/hg19; chr11:64,136,074-64,136,176) of the RPS6KA4 gene (GRCh37/hg19; chr11:64,126,625-64,139,687). We chose this non-canonical miRNA because of the very low read counts in small RNA-seq datasets (miRBase release 21), to further assess the sensitivity of the method. The full intron sequence was used as bait and prepared according to the procedure described herein. To further assess the possibility to downscale the assay, we used as little as 10 ng of purified small RNAs. As shown in Figure 3, we were successful in capturing and identifying the mature miRNA sequence from very low amounts of RNA.

## 3. Discussion

The reason for developing yet another technical approach to uncover small RNAs stemmed from the realization that if alternative pathways in the production of small regulatory RNAs exist, then the analysis of RNA-seq data is complicated by the lack of predictive tools and basic data about their characteristics. In addition, several reports suggest that the large majority of mature miRNAs are expressed at very low levels in eukaryotic cells [25,26,27,28]. We therefore aimed at developing a method free of *a priori* to identify, clone and sequence even weakly expressed new miRNAs, although it implies to delimit a genomic region of interest already implicated in normal development or disease for instance. We list below the main clear benefits offered by psRNA-seq over high-throughput approaches:

(1) psRNA-seq is a highly sensitive technique that can detect even low amounts of mature miRNAs (or any other short RNA), as exemplified by the amplification of the weakly expressed has-miR-21 star and hsa-miR-1237, that would not pass low-thresholding or elimination of isolated reads applied to eliminate high-throughput sequencing errors [16,17,18,19]. We can infer from our data that psRNA-seq is a method equally sensitive to high-throughput sequencing for an already known miRNA, highly expressed in the relevant cellular system. Obviously, no conclusion can be drawn regarding yet unidentified small RNAs.
(2) psRNA-seq does not require knowing the biogenesis pathway of the small RNA of interest. Therefore, not only miRNA, but also mirtrons, simtrons, snoRNAs, snRNAs, and others small ncRNAs can potentially be detected and cloned using psRNA-seq provided the genomic location, even approximate, of the sequence containing the small RNA is known. It is indeed simple to refine the position using overlapping sequences as we have done with hsa-miR-21. We used 60 to 102 nt long baits to minimize secondary structures that may form, although we have experience of RNA pull down assays using much longer bait RNAs, which efficiency has to be tested individually [29,30].
(3) psRNA-seq can be performed on rare samples like adult stem cells or patient biopsies since it does not require large amounts of material. While microarrays and RNA deep sequencing needs are in the range of several micrograms of sample RNA, we used as less as 10 ng of fractionated RNA, an amount that can be further reduced. Indeed, from all the RNA samples that we prepared, we used 1/100th by PCR reactions and obtained thousands of colonies after bacterial transformation, clearly suggesting that 0.1 ng of fractionated RNA should be enough to obtain similar results (0.1 ng of small fractionated RNA represents about 1 ng of total RNA and as little as 100 human cells).
(4) psRNA-seq is a simple, rapid, and inexpensive method to set up. It does not require special skills in molecular biology or bioinformatics. Indeed, it does not require long and tricky procedures to prepare libraries to be sequenced by RNA-seq technologies or challenging bioinformatical analysis of large datasets.
(5) psRNA-seq is highly flexible. It is not specific to miRNAs (canonical and from intron origin) and can also be used to capture plenty of other ncRNAs such as snRNAs, snoRNAs and many other still-unknown small RNAs. The method is also easily transposable to other species, with some restrictions (see below).

We should mention some limitations to the technique. First, psRNA-seq relies on the initial knowledge of the genomic sequence; therefore, it cannot be used for discovering small RNAs in species with unannotated genomes. Second, psRNA-seq was designed as a qualitative method to capture RNA with unknown sequences and thus, does not allow a quantitative analysis. However, once captured and sequenced following psRNA-seq, mature miRNAs can be quantified using classical stem-loop RT-qPCR or other techniques [23,31,32] and commercially available kits such as miR-X from Clontech (Paris, France),MystiCq miRNA from Sigma (Paris, France), QuantimiR from System Biosciences (Paris, France). Third, psRNA-seq will be difficult to adapt to high-throughput screening. Finally, since psRNA-seq is based on a polyadenylation reaction at the 3′-end of the captured RNA, it will not be suited to the capture of miRNAs from plants or piwi interacting RNAs (piRNAs). Indeed, plant miRNAs, as well as piRNAs, possess a 2′-O-methyl modification on the ribose of their 3′ extremity [31,32,33] which prevents the addition of nucleotides to the 3′-end and therefore the activity of the poly(A) polymerase [32,33].

In support of the use of psRNA-seq, this method recently allowed us to identify and clone a novel mirtron produced by a human gene in specific developmental conditions (in preparation; to be published elsewhere) and from another pathogenic locus is disease conditions (in preparation; to be published elsewhere).

## 4. Material and Methods

Detailed experimental protocol is given in Supplementary Data 1 and a scheme of the pocket-sized RNA-seq method is shown in Figure 1.

### 4.1. Bait Preparation

The first step requires the production of *in vitro* transcribed RNA from the genomic region of interest that will be used as bait. We chose to use MessageMuter™ shRNA production kit (EpiCentre, Chicago, IL, USA) to produce RNAs complementary to the human precursor of hsa-miR-21, or the irrelevant luciferase gene as a negative control, according to the manufacturer’s instructions and as previously described [30]. We chose the extensively studied hsa-miR-21, that has been described as a proto-oncomiR associated with a wide variety of cancers, so that we could use untransformed primary myoblast cells as negative controls [34,35,36]. The size of the three different baits that we used ranged from 60 to 90 bp (Figure S3A) and sequences are indicated in Supplementary Materials 1. For reasons that will be explained below, RNAs need to be first dephosphorylated. One microgram of bait RNA was therefore dephosphorylated using 1 U CIP (Alkaline Phosphatase, Calf Intestinal, New England Biolabs, Evry, France) at 37 °C for 60 min and RNAs were purified by phenol-chloroform extraction.

### 4.2. RNA Sample Preparation

RNA samples that will be hybridized to the bait were isolated from MCF-7 breast cancer cells or primary myoblasts with TRI reagent (Sigma) as previously described [30,37]. To reduce background and non-specific RNA binding, we size-fractionated RNAs using mirVana™ miRNA Isolation Kit (Life Technologies, Carlsbad, CA, USA), to retain miRNAs (mature and pre-mature) contained in the flow-through, according to manufacturer’s instructions (Figure S3B).

### 4.3. Hybridization of the Bait with Size-fractionated Short RNAs

After phenol/chloroform extraction, 400 pmol of RNA bait were denature at 95 °C for 2 min and placed on ice for an additional 2 min. RNA was then annealed to 500 pmol of a specific biotinylated oligonucleotide (sequences available at the end of this subsection) in 50 mM KCl, 1 U/mL RNaseOUT (Life Technologies) for 1h at RT. Streptavidin magnetic beads (Dynabeads, Life Technologies), prewashed twice in 0.1 M NaOH, 0.05 M NaCl and once in 0.1 M NaCl, were incubated with the RNA/oligonucleotide complex in binding/washing buffer (10 mM Tris–HCl pH 7.5, 1 mM EDTA, 1 M NaCl) for 30 min at RT. Pre-incubated beads were washed twice in binding/washing buffer and added to 100 ng of small purified RNAs (<200 nt), as described above, for an additional 1 h at RT. “Small RNA/RNA bait/magnetic beads” complexes were washed twice with RNA binding buffer (10% Glycerol, 10 mM Hepes, 150 mM KCl, 1 mM EDTA, 0.5% Triton X100) adjusted to 300 mM KCl and once with RNA binding buffer.

### 4.4. Polyadenylation of the Captured RNAs

After phenol/chloroform extraction, 1 U of poly (A) Polymerase (Life Technologies) was then used to add a poly(A) tail to the recovered RNAs following the manufacturer’s instructions, along which, 1 h incubation generally adds between 50 and 200 A at the 3′-ends. Polyadenylated RNAs were then purified by phenol-chloroform extraction.

### 4.5. Adapted RACE-PCR Amplification of the Captured Products

We then performed full RACE-PCR using the GenRacer kit (Life Technologies) as previously published [14,15] with slight modifications. Briefly, the protocol started at the ligation procedure. Because the baits were first dephosphorylated (see Subsection 2.1) and the oligonucleotides used for pull-down were biotinylated at their 5′-end, the only sequences that can be ligated to adaptors and further amplified are the small RNAs associated and trapped with the bait RNAs. Polyadenylated RNAs were then reverse transcribed using the kit adaptor which contains a poly(T) sequence in 3′-end to give a product described in Figure 1. After PCR amplification (Figure S1C) and cloning into TOPO-TA vector (Life Technologies), sequences of the so purified RNAs were retrieved by sequencing of both strands (MWG Eurofins, Ebersberg, Germany).

### 4.6. Stem-Loop RT-qPCR

Stem-loop RT-qPCR was performed as previously described [23] using stem-loop primers designed with miRNA Primer Design Tool Website (http://mirnadesigntool.astridresearch.com). All reactions, including no-template controls and RT minus controls, were run in duplicate. Normalization was performed relative to the signal of RNU6-1 amplification. Stem-loop primers for reverse transcription of miR-10a and miR-21 were 5′-GTT GGC TCT GGT GCA GGG TCC GAG GTA TTC GCA CCA GAG CCA ACC ACA AA-3′ and 5′-GTT GGC TCT GGT GCA GGG TCC GAG GTA TTC GCA CCA GAG CCA ACT CAA CA-3′, respectively. For qPCR, forward primers were 5′-TGT TTT TTT TTA CCC TGT AGA TCC GAA-3′ and 5′-GTT TGG TAG CTT ATC AGA CTG A-3′, for miR-10a and miR-21, respectively, while reverse primer was universal primer (5′-GTG CAG GGT CCG AGG T-3′). U6 forward and reverse primers were 5′-CTC GCT TCG GCA GCA CA-3′ and 5′-AAC GCT TCA CGA ATT TGC GT-3′, respectively.

### 4.7. RNA-seq Datasets Used

Genome-wide RNA-Seq data sets used here were generated by Chris Burge’s lab at the Massachusetts Institute of Technology (Cambridge, MA, USA) and by Roderic Guigó’s lab at the Center for Genomic Regulation (CRG, Barcelona, Spain) published by Wang *et al*. (2008), and deposited to the Short Read Archive section of GEO at NCBI under accession number GSE12946 [36], and by Hannon and Gingeras laboratories members at the Cold Spring Harbor Laboratory (Cold Spring Harbor, NY, USA) using ENCODE cell lines (GSE24565) [38,39,40]. Data referenced in miRBase were also used herein, especially to compare the ratios obtained between guide and star mature miR-21 [35,37,39,41].

## Supplementary Files

Supplementary File 1
