# Supplementary material for: “Pocket-sized RNA-Seq”: A Method to Capture New Mature microRNA Produced from a Genomic Region of Interest
Source: Noncoding RNA. 2015 Jul 3;1(2):127–38. doi: 10.3390/ncrna1020127 (PMC5932543; doi:10.3390/ncrna1020127)
Supplement: Supplementary File 1 [file ncrna-01-00127-s001.pdf]

Non-Coding RNA **2015**, *1*, 127-138; doi:10.3390/ncrna1020127

Supplementary Materials

S1. Bait Preparation

Materials

- MessageMuter™ shRNA production kit (Cat. No. MM031110, Epicentre, Chicago, IL, USA)
- Alkaline Phosphatase, Calf Intestinal (CIP, Cat. No. M0290S, New England Biolabs, Evry, France)
- Bait1, hsa-miR-21, chr17:57918611-57918674 (hg19); stock solution 100 µM (Eurofins, Germany)

  5′-TTGCCATGAGATTCAACAGTCAACATCAGTCTGATAAGCTACCC*GACAAGG TGGTAC AGCCATG*-3′. The mature guide mir-21 sequence is underlined (simple) and complementary sequence of the biotinylated oligonucleotide is italicized.

- Bait2, hsa-miR-21, chr17:57918611-57918697 (hg19) ; stock solution 100 µM (Eurofins)

  5′- GTCAGACAGCCCATCGACTGGTGTTGCCATGAGATTCAACAGTCAACATCA GTCT GATAAGCTACCC*GACAAGGTGGTACAGCCATG*-3′. The mature guide and star mir-21 sequences are underlined (simple and double, respectively) and complementary sequence of the biotinylated oligonucleotide is italicized.

- Bait3, hsa-miR-21, chr17:57918648-57918722 (hg19) ; stock solution 100 µM (Eurofins)

  5′-ATGGTCAGATGAAAGATACCAAAATGTCAGACAGCCCATCGACTGGTGTTG CCA *TGAGATTCAACAGTCAACATC*-3′. The mature star mir-21 sequence is underlined (double) and complementary sequence of the biotinylated oligonucleotide is italicized.

- Bait4, Control bait (luciferase); stock solution 100 µM (Eurofins)

  5′-AAGGCTTACGCTGAGTACTTCGACTTGCTTCTCGAAGTACTCAGCGTAAGT ATAG TGA-3′.

- CIP (Alkaline Phosphatase, Calf Intestinal, New England Biolabs)
- RNase-free 1.5 mL or 0.5 mL polypropylene microfuge tubes
- RNase-free water
- Adjustable pipettors
- RNase-free tips
- Heat blocks (Eppendorf, Montesson, France)
- NanoDrop spectrophotometer (Thermo Scientific, Courtaboeuf, France)
- Microcentrifuge (14,000 × g)

1. Produce bait RNAs following the MessageMuter™ shRNA production kit protocol. The final RNA pellet was resuspended in 20 µL of the 5X annealing buffer (provided in the kit) and 80 uL of RNase-free water.

   Hints: The region of interest used as a bait can also be cloned in a vector under the control of a T7 promoter, PCR amplified or directly synthesized including the T7 promoter sequence.

Hints: Shorter is better; if the region producer of the miRNA is not known, it is better to use several overlapping shorter RNAs as baits than a longer one which will present highly structured RNA domains and will be more difficult to denature.

2. Concentration was first determined by A_{260} using NanoDrop spectrophotometer and further evaluated for appropriate size and absence of degradation by polyacrylamide gel (protocol from the MessageMuter™ shRNA production kit).
3. Mix 1 U CIP with 1 µg bait RNA in a 20 µL final volume.
4. Incubate at 37 °C for 1h.
5. RNAs were purified by phenol-chloroform extraction (see PHENOL-CHLOROFORM EXTRACTION section).

S2. RNA Sample Preparation

Materials

- MCF-7 breast cancer cells (Cat. No. HTB-22, ATCC)
- Primary myoblasts (From Dr. Vincent Mouly, Myology Institute, Paris, France)
- TRI reagent (Cat. No. T9424, Sigma)
- 100% and 70% ethanol
- mirVana™ miRNA Isolation Kit (Cat. No. AM1560, Life Technologies, Courtaboeuf, France)
- RNase-free 1.5 mL or 0.5 mL polypropylene microfuge tubes
- RNase-free water
- Adjustable pipettors
- RNase-free tips
- NanoDrop spectrophotometer (Thermo Scientific)
- Microcentrifuge (14,000 × g)

1. RNA samples were isolated from PBS-washed 10^6 cells using TRI reagent accordingly to manufacturer's instructions. RNA pellet was resuspended in 50 µL of RNase-free water.
2. Concentration was determined by A_{260}.
3. Small RNAs were then size-fractionated using mirVana™ miRNA Isolation Kit accordingly to manufacturer's instructions and further evaluated for appropriate size and absence of degradation by polyacrylamide gel (protocol from the MessageMuter™ shRNA production kit).

Hints: The mirVana™ miRNA Isolation Kit proposed isolation of small RNA from cell lines using one unique protocol. However, we observed that small RNAs purified from total RNAs (themselves formerly extracted using Trizol or TRI reagent) were cleaner in a two-step procedure.

S3. Bait and RNA Targets Hybridization

Materials

- Dephosphorylated RNA baits from BAIT PREPARATION section
- 5′-biot-CATGGCTGTACCACCTTGTC-3′ to pull down bait1 and bait2; stock solution 500 µM (Eurofins)

- 5′-biot-GATGTTGACTGTTGAATCTCA-3′ to pull down bait3; stock solution 500 µM (Eurofins)
- 5′-biot-TCACTATACTTACGCTGAGTAC-3′ to pull down luc; stock solution 500 µM (Eurofins)
- Binding/Washing buffer 1x; BW1x (see S7.1)
- Binding/Washing buffer 2x; BW2x (see S7.2)
- RNA Binding Buffer 1; RBB1 (see S7.3)
- RNA Binding Buffer 2; RBB2 (see S7.4)
- RNA Binding Buffer 3; RBB3 (see S7.5)
- Wash 1 buffer; W1 (see S7.6)
- Wash 2 buffer; W2 (see S7.7)
- RNaseOUT (Cat. No. 10777-019, Life Technologies, Carlsbad, CA, USA)
- Dynabeads® M-280 Streptavidin (Cat. No. 11205D, Dynabeads, Life Technologies)
- RNase-free water
- RNase-free 1.5 mL or 0.5 mL polypropylene microfuge tubes
- Adjustable pipettors
- RNase-free tips
- Heat blocks (Eppendorf)
- Rotating wheel
- Magnets

1. Denature 400 pmol of RNA bait (in 10 µL RNase-free water) at 95 °C for 10 min and place on ice immediately for an additional 5 min.

   Hints: Because of the small volume, denaturation using a thermal cycler with a heated lid would reduce evaporation.

2. Add 500 pmol of a specific biotinylated oligonucleotide (in 10 µL RNase-free water) and complete at 250 µL with RBB1.

   Hints: For some RNAs, it might be more efficient (less background) to first hybridize biotinylated oligonucleotides and streptavidin magnetic beads, then to add in vitro transcribed RNA.

3. Incubate 1 h at RT on rotating wheel.
4. During the incubation time, wash the M-280 magnetic beads twice in W1 and once in W2. Dry beads are resuspended in 250 µL BW2x.
5. Mix RNA bait/biotinylated oligonucleotide (from 3.) to M-280 magnetic beads (from 4.) and incubate at RT for 30 min to form a complex RNA bait/Biotinylated oligonucleotide/M-280 magnetic beads (RBM complex).

   Hints: For some RNAs, it might be more efficient (less background) to first hybridize biotinylated oligonucleotides and streptavidin magnetic beads, then to add in vitro transcribed RNA.

6. Wash RBM twice with BW1x.
7. Incubate 100 ng of small purified RNAs (from RNA SAMPLE PREPARATION section) with RBM in 500 µL BW1x for 1 h at RT.

Hints: We used as little as 10 ng of small fractionated RNAs which correspond to approximately 100 ng total RNAs. We however did not adapt and reduce the amount of bait, biotinylated oligonucleotide and beads to this small amount.

8. Coated beads were washed twice in RBB3 and once in RBB2.
9. Elute small RNAs captured by RBM with 100 µL RNase-free water. Incubate at 95 °C for 2 min, then place the tube on the magnet and quickly transfer the supernatant containing the RNAs to a new RNase-free tube.
10. Small RNAs were purified by phenol-chloroform extraction (see PHENOL-CHLOROFORM EXTRACTION section).

S4. Polyadenylation

Materials

- Captured small RNAs from BAIT AND RNA TARGETS HYBRIDIZATION section
- *E. coli* Poly(A) Polymerase (Cat. No. M0276S, New England Biolabs)
- RNase-free 1.5 mL or 0.5 mL polypropylene microfuge tubes
- RNase-free water
- Adjustable pipettors
- RNase-free tips
- Heat blocks (Eppendorf)

1. Incubate captured small RNAs and 1 U of poly(A) polymerase in manufacturer’s buffer for 1 h at 37 °C with 1mM ATP.

   Hints: Shortened poly(A) polymerase incubation will of course shorten addition of A at the 3’-tail, but we do not recommend it as we did not amplify anything at a incubation time of 15 min. Indeed, the 1h incubation time added from 19 to 38 A as checked after sequencing.

2. Polyadenylated captured small RNAs were purified by phenol-chloroform extraction (see PHENOL-CHLOROFORM EXTRACTION section).

S5. Adaptor Ligation and Amplification

Materials

- Polyadenylated captured small RNAs from POLYADENYLATION section
- GenRacer kit (Cat. No. L1500-01, Life Technologies)
- GeneJET Gel Extraction Kit (Cat. No. K0691, Thermo Scientific, Waltham, MA, USA)
- 100% and 70% ethanol
- RNase-free water
- RNase-free 1.5 mL or 0.5 mL polypropylene microfuge tubes
- Adjustable pipettors
- RNase-free tips
- Heat blocks (Eppendorf)

- Microcentrifuge (14,000 × g)
- Thermocyler

1. Denature polyadenylated captured small RNAs at 65 °C for 5 min and immediately place on ice to chill for 2 min.
2. Add 6 µL of denatured captured small RNAs, 1 µL of 10X ligase buffer, 1 µL of 10 mM ATP, 1 µL of RNaseOUT (40 U) and 1 µL of T4 RNA ligase (all provided in GeneRacer kit) to the pre-aliquoted and lyophilized GeneRacer™ RNA Oligo and incubate at 37 °C for 1 h.

   Hints: You can use your own RNA adapters. Moreover, a DNA adaptor can be used at 5′-end but the reverse transcription will also be less efficient.

3. Ligation products were purified by phenol-chloroform extraction (see PHENOL-CHLOROFORM EXTRACTION section).
4. Add 1 µL of GeneRacer oligo dT primer (50 µM) and 1 µL of dNTP (10 mM each) mix (provided in the kit) and incubate at 65 °C for 5 min.
5. Place on ice immediately to chill for 2 min.
6. Add 4 µL of 5X RT buffer, 15 U of AMV RT, 40 U of RNaseOUT and 2 µL of RNase-free water (all provided in GeneRacer kit) and incubate at 45 °C for 15 min.
7. Incubate at 85 °C for 15 min and spin briefly.
8. Amplify 1/100th of RT-PCR products by PCR using GeneRacer 5′ and 3′ primers (provided in the kit) using manufacturer's thermocycling and run 10–20 µL of the PCR product on 1% agarose gel + ethidium bromide.

   Hints: It is sometimes useful to perform an additional nested PCR using internal primers.

9. Clone PCR products in pCR4-TOPO vector provided in the GeneRacer kit and sent for sequencing several clones.

S6. Phenol-chloroform Extraction

Materials

- Phenol:CHCl3:isoamyl alcohol, 25:24:1, saturated with 10 mM Tris Cl, pH 8.0/1 mM EDTA (Cat. No. P3803, Sigma)
- $CHCl_3$ (Cat. No. 25668, Sigma)
- 3 M sodium acetate pH 5.5
- Glycogen (Cat. No. AM9510, Life Technologies)
- 100% and 70% ethanol
- RNase-free water
- RNase-free 1.5 mL or 0.5 mL polypropylene microfuge tubes
- Adjustable pipettors
- RNase-free tips
- Microcentrifuge (14,000 × g)

1. Adjust volume to 100 µL with RNase-free water.

2. Add 100 µL phenol:chloroform:isoamyl alcohol 25:24:1 and vortex vigorously for 30 s.
3. Centrifuge at maximum speed for 5 min and transfer aqueous phase (top) to a new tube.
4. Add 1 µL glycogen, 10 µL 3 M sodium acetate pH 5.5, 220 µL 100% ethanol and vortex briefly.
5. Place on ice for 10–20 min.
6. Centrifuge at maximum speed for 30 min at +4 °C and remove the supernatant carefully.
7. Wash with 500 µL 70% ethanol and centrifuge at maximum speed for 2 min at +4 °C.
8. Carefully remove the ethanol by pipet and air-dry the pellet for no more than 1–2 min at room temperature.
9. Resuspend the pellet in the appropriate volume of RNase-free water according to the step of protocol.

S7. Reagents and Solutions

Use deionized, distilled water in all recipes.

S7.1. Binding/Washing buffer 1x; BW1x

5 mM Tris HCl
0.5 mM EDTA
1 M NaCl
120 U/mL RNaseOUT
Add RNaseOUT extemporaneously.

S7.2. Binding/Washing buffer 2x; BW2x

10 mM Tris HCl
1 mM EDTA
2 M NaCl
120 U/mL RNaseOUT
Add RNaseOUT extemporaneously.

S7.3. RNA Binding Buffer 1; RBB1

50 mM KCl
120 U/mL RNaseOUT
Add RNaseOUT extemporaneously.

S7.4. RNA Binding Buffer 2; RBB2

10% Glycérol
10 mM Hepes
150 mM KCl
1 mM EDTA
0.5% Triton X100
120 U/mL RNaseOUT

Add RNaseOUT extemporaneously.

S7.5. RNA Binding Buffer 3; RBB3

10% Glycérol
10 mM Hepes
300 mM KCl
1 mM EDTA
0.5% Triton X100
120 U/mL RNaseOUT
Add RNaseOUT extemporaneously.

S7.6. Wash 1 buffer; W1

0.05 M NaCl
0.1 M NaOH

S7.7. Wash 2 buffer; W2

0.1 M NaCl

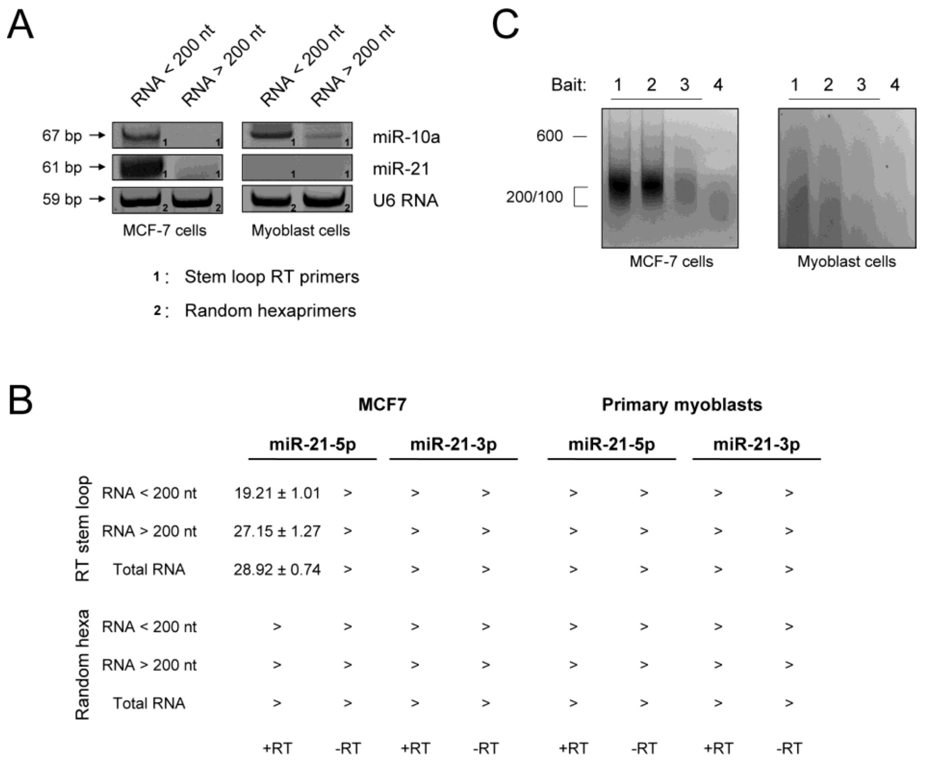

Figure S1. (**A**) Stem loop RT-PCR was used to evaluate the presence of a control miRNA (hsa-mir10a) and of the miRNA processed from hsa-primiR-21 (miR-21). Mature miRNA-specific primers were designed using miRNA Primer Design Tool Website and normalization was performed relative to the signal of RNU6-1 amplification (U6 RNA). **(B)** Ct obtained by stem-loop RT-qPCR using RNAs purified (short and long RNAs, < and > 200 nt, respectively, and total RNA) from MCF-7 and primary myoblast cells, and using stem-loop or random hexamer primers, with or without reverse transcriptase. Results are the mean ± standard deviation of at least 3 experiments per conditions performed in duplicates. -5p and -3p, guide and star mature miR-21 strand, respectively; >, Ct were > 35 cycles; +RT and –RT, with or without reverse transcriptase, respectively. **(C)** Agarose gel of the PCR amplification of the final product. PCR products from reverse transcribed RNAs isolated and modified as described in the material and methods section, using 3 different baits related to miR-21 sequence (1-3) and one corresponding to the luciferase sequence. Left panel, psRNA-seq performed using MCF-7 cell line; right panel, psRNA-seq performed using primary cells (human myoblasts).

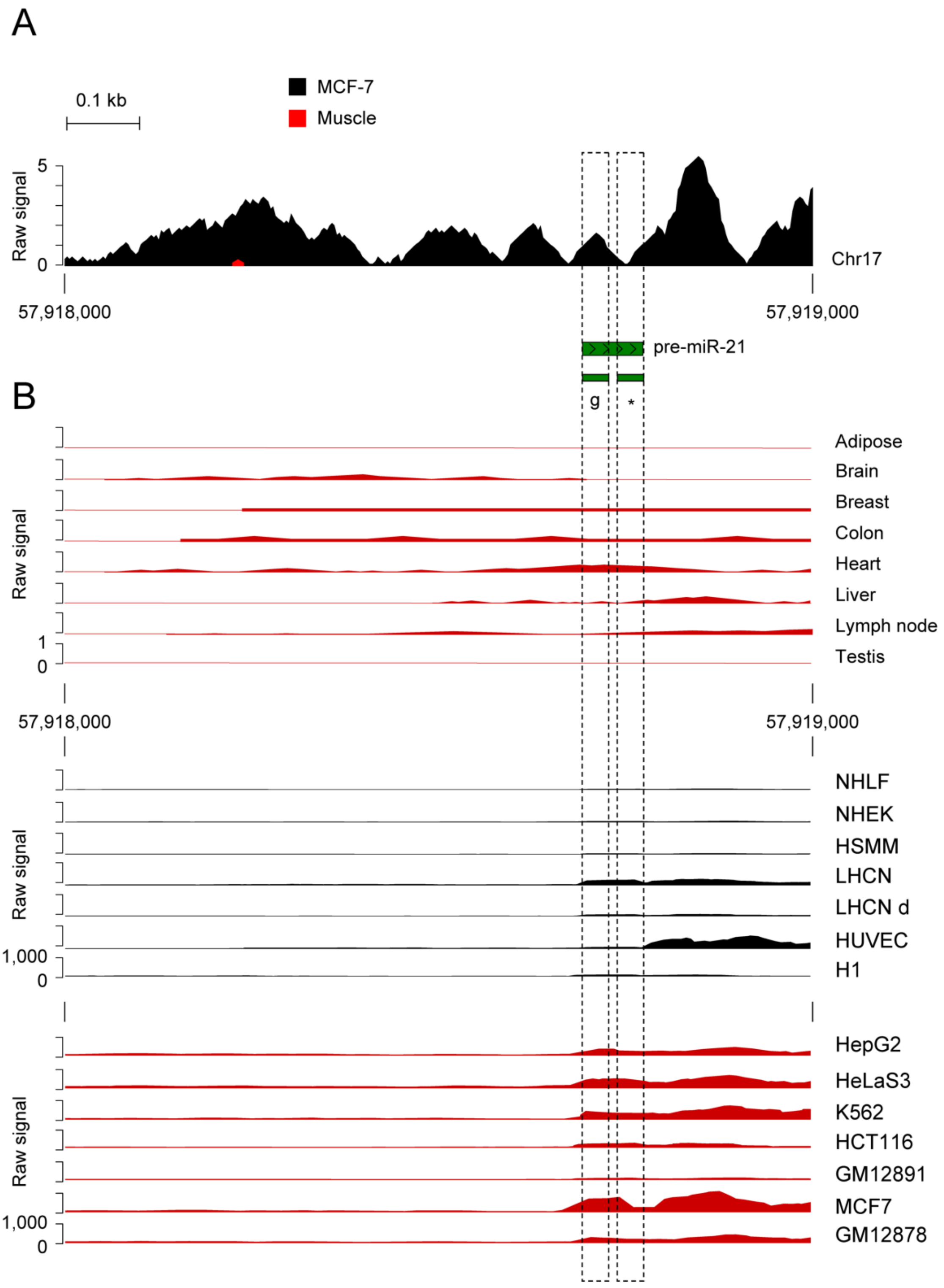

Figure S2. (A) Schematic representation of RNA-seq data amongst the hsa-miR-21 locus using data generated by Chris Burge's lab and by Hannon's and Gingeras's laboratories as described in the main text. Comparison of RNA-seq raw data in MCF-7 cells (black) and normal muscle tissue (red) data. Both guide (g) and star (*) mature sequences are given in green. **(B)** as in (A), RNA-seq raw data amongst the miR-21 locus in normal tissue (first set, Top panel), normal cell lines (second set, middle panel) and cancer cells (third set, bottom panel). For more information about ENCODE cell lines, see http://genome.ucsc.edu/encode/cellTypes.html.

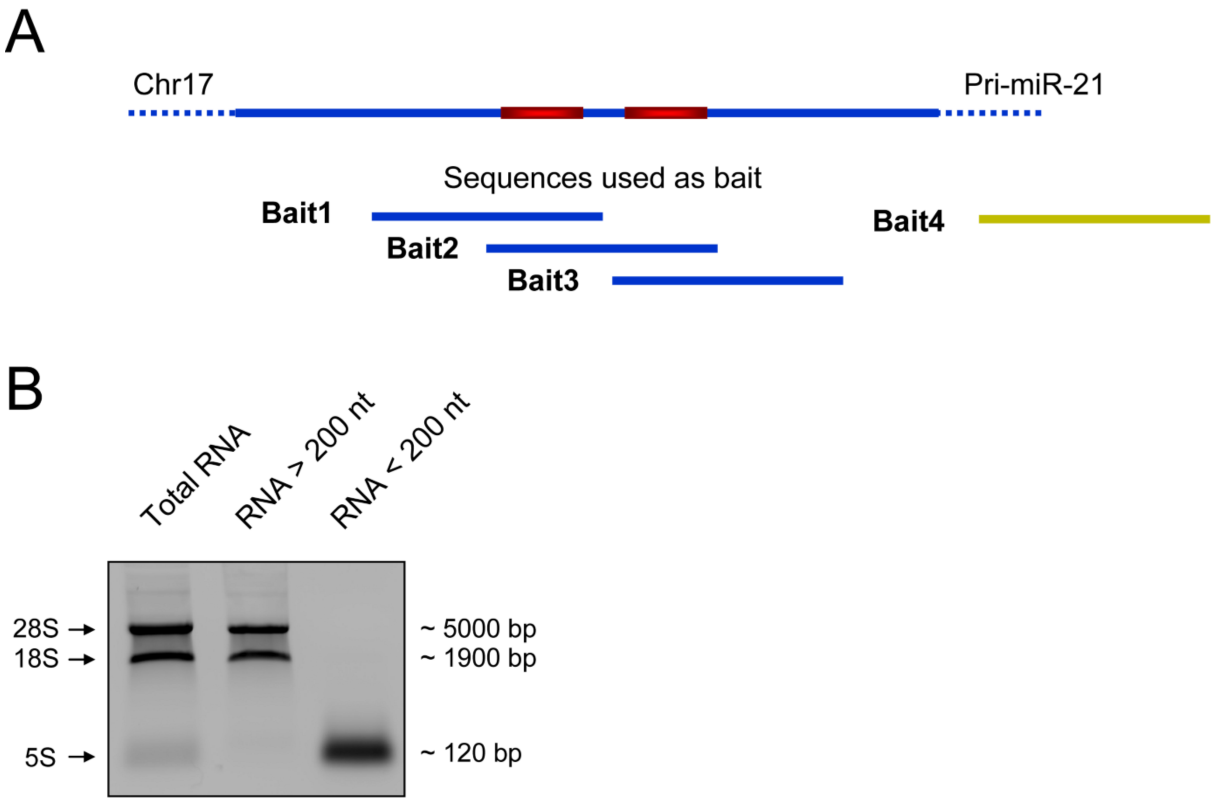

Figure S3. (A) Schematic representation of the position of the baits related to the pri-miR-21 locus. **(B)** Short and long RNAs, < and > 200 nt, respectively, were separated using mirVana™ miRNA Isolation Kit (Life Technologies). Agarose gel was used to control purity and quality of 1 µg of each RNA samples.

© 2015 by the authors; licensee MDPI, Basel, Switzerland. This article is an open access article distributed under the terms and conditions of the Creative Commons Attribution license (http://creativecommons.org/licenses/by/4.0/).
